# The Additive Value of Cardiovascular Magnetic Resonance in Convalescent COVID-19 Patients

**DOI:** 10.3389/fcvm.2022.854750

**Published:** 2022-04-07

**Authors:** Alessandra Borlotti, Helena Thomaides-Brears, Georgios Georgiopoulos, Rajarshi Banerjee, Matthew D. Robson, Dahlene N. Fusco, Pier-Giorgio Masci

**Affiliations:** ^1^Perspectum Ltd., Oxford, United Kingdom; ^2^School of Biomedical Engineering and Imaging Sciences, King’s College London, London, United Kingdom; ^3^Department of Clinical Therapeutics, National and Kapodistrian University of Athens, Athens, Greece; ^4^Tulane University School of Medicine, New Orleans, LA, United States

**Keywords:** cardiovascular magnetic resonanace, long-COVID, myocardial injury, multi-organ damage, convalescent COVID-19 patients

## Abstract

In COVID-19 the development of severe viral pneumonia that is coupled with systemic inflammatory response triggers multi-organ failure and is of major concern. Cardiac involvement occurs in nearly 60% of patients with pre-existing cardiovascular conditions and heralds worse clinical outcome. Diagnoses carried out in the acute phase of COVID-19 rely upon increased levels of circulating cardiac injury biomarkers and transthoracic echocardiography. These diagnostics, however, were unable to pinpoint the mechanisms of cardiac injury in COVID-19 patients. Identifying the main features of cardiac injury remains an urgent yet unmet need in cardiology, given the potential clinical consequences. Cardiovascular magnetic resonance (CMR) provides an unparalleled opportunity to gain a deeper insight into myocardial injury given its unique ability to interrogate the properties of myocardial tissue. This endeavor is particularly important in convalescent COVID-19 patients as many continue to experience chest pain, palpitations, dyspnea and exertional fatigue, six or more months after the acute illness. This review will provide a critical appraisal of research on cardiovascular damage in convalescent adult COVID-19 patients with an emphasis on the use of CMR and its value to our understanding of organ damage.

## Introduction

Most people affected by severe acute respiratory syndrome coronavirus 2 (SARS-CoV-2) infection are asymptomatic or experience milder symptoms. During the early phases of the pandemic ∼15% of patients required hospitalization, and up to 5% needed treatments in intensive care because of severe coronavirus disease 2019 (COVID-19) (1). Globally, these numbers have decreased as a result of vaccination. For example, in January 2022 the hospitalization rate in the United Kingdom (UK) decreased to 3.7% in vaccinated patients and 7% in the overall population (2, 3). However, these figures may change again as new viral variants arise (e.g., omicron) requiring higher levels of population immunity (4). Although the predominant manifestation of COVID-19 is viral pneumonia, COVID-19 also causes cardiovascular disorders including acute myocardial injury, arrhythmias, and thromboembolism (5). Cardiovascular involvement is a strong independent predictor of in-hospital mortality from COVID-19 (6, 7). In most instances, cardiovascular involvement was revealed at hospital admission, by chest pain or elevated circulating biomarkers [cardiac troponins (cTn) or natriuretic peptides], and co-segregated with disease severity, increased inflammatory markers, advanced age, and cardiovascular risk factors (7–9).

Compelling recent evidence indicates that convalescent COVID-19 patients have a heightened risk of cardiovascular disease at one year after the initial infection irrespective of the severity of COVID-19 at the acute stage (10). Some patients experience long-lasting symptoms and complications from COVID-19 infection, referred to as *long covid or post-COVID syndrome or long-haul COVID*. This is a heterogeneous, yet poorly classified and understood condition that is characterized by long-lasting and fluctuating symptoms persisting after the initial infection dissipates (11, 12). A recent systematic review comprising 57 studies and more than 250,000 COVID-19 survivors, showed that 54% of the survivors continue to experience at least one symptom after six or more months (13). According to the UK’s Office for National Statistics, 1.2 million people in the UK experienced long covid between April 2020 and October 2021, of which about two-thirds and one-third of patients still have symptoms beyond three and 12 months, respectively (14, 15). In the United States, a study of 2 million COVID-19 survivors identified that 23% of convalescent patients had continued symptoms beyond four weeks, and, as also found in the UK (16), represented all age groups and included patients whose initial SARS-CoV-2 infection was asymptomatic or mildly symptomatic (15, 17, 18). Symptoms related to cardiovascular autonomic dysfunction seem to be common in patients affected by long COVID syndrome (19). Moreover, pre-existing cardiovascular risk factors, socio/economic disparities, and comorbidities concur in amplifying the adverse, long-term consequences of COVID-19 (syndemic) (20–23).

Elucidating the long-term clinical consequences of COVID-19 mediated cardiac injury is thus, an urgent, yet unmet need in cardiology. Cardiovascular magnetic resonance (CMR) can pioneer in this area given its unparalleled capability in assessing cardiovascular structure and function, alongside accurate, precise and in-depth tissue characterization of the myocardium. This review will provide a critical appraisal of the main research on cardiovascular damage in convalescent adult COVID-19 patients, emphasising the use of CMR and its value to our understanding of organ damage in these patients.

## Pathophysiology and Epidemiology of Cardiovascular Damage in Acute COVID-19

Although predominantly a respiratory disease, the acute phase of COVID-19 can affect multiple organs particularly in patients with moderate to severe infection and pre-existing comorbidities (24–26). In this context, cardiovascular involvement has gained attention given its relatively high incidence in hospitalized COVID-19 patients and the negative impact on clinical outcome (6, 7). Myocardial injury, usually defined as elevation of high-sensitivity cTn above the 99th percentile of the upper reference limit, has been documented in 20–30% of patients hospitalized with COVID-19, with a prevalence up to 55–62% in patients with pre-existing cardiovascular conditions (6, 7). The virus can cause direct or indirect cardiovascular damage, including myocarditis-like injury, acute thrombo-embolic disease of the coronary (acute coronary syndrome) or pulmonary arterial (pulmonary embolism) circulation, arrhythmias, and cardiogenic shock (27, 28). The mechanisms of myocardial injury remain unclear given that endomyocardial biopsy or CMR were seldom carried out in hospitalized patients. The proposed mechanisms for SARS-CoV-2-mediated cardiac injury include direct effects on cardiomyocytes or endothelial cells, indirect effects due to hypercoagulability and injury resulting from cytokine storm or autoimmunity (29).

However, autopsy series indicate that lymphocytic myocarditis is uncommon, while infiltration of the myocardium with pro-inflammatory macrophages along with multi-thrombi in the coronary circulation are frequent (9, 30–32). Lymphocytic myocarditis was observed in only 1.4% of a pooled series of 277 patients who died from COVID-19 (33); the virus particles were reported in the cardiac interstitial cells, but not in the cardiomyocytes, in 24 out of 39 consecutive autopsies (30). Altogether, these findings suggest underlying diffuse myocardial inflammation, microvascular thrombi and scattered cardiomyocyte necrosis as the common histopathological features of cardiovascular involvement in acute COVID-19.

## Cardiovascular Damage in Convalescent COVID-19 Patients

Concerns exist for long-term cardiovascular abnormalities in convalescent patients. In a study of nearly 1,300 patients with SARS-CoV-2 infection followed-up after hospitalization, half of the patients had at least one symptom at 12-month follow-up, and 30%, 9%, and 7% of those complained of persisting dyspnea, palpitations and chest pain, respectively (20). Unquestionably, cardiac biomarkers, namely cTn and natriuretic peptides, and transthoracic echocardiography (TTE) play a major role in unstable, hospitalized COVID-19 patients. Nearly two-third of patients with elevated cTn showed abnormalities at TTE including left ventricle wall motion abnormalities (24%) or global dysfunction (18%)(34). In a series of more than 1,200 hospitalized patients with COVID-19, TTE unveiled LV abnormalities in 39% of patients, however, without pinpointing the underlying cause in many patients (35). Moreover, a recent meta-analysis indicates that right ventricular (RV) dysfunction due to pulmonary and direct RV injury occurs in one out of five COVID-19 patients and is associated with all-cause mortality (36).

Infection control policy and logistic hurdles limited the use of CMR for investigating cardiac injury in the acute phase of COVID-19 (22), but there is a rapidly growing body of evidence substantiating the value of CMR in convalescing patients that continue to have symptoms (Supplementary Table 1). This imaging modality enables an accurate and precise quantification of the ventricular volumes and systolic function alongside in-depth tissue characterization of the myocardium. CMR is key in studying RV remodeling and dysfunction which is an important prognosticator in COVID-19 patients (37). Late gadolinium enhancement (LGE) is a well-established technique to identify and measure replacement fibrosis (scarring of the myocardium), which is complemented by T1-mapping and T2-mapping techniques. The latter measure the composition of myocardium (e.g., free-water content or interstitial fibrosis) by interrogating changes in its intrinsic relaxivity properties that can change with myocardial injury (38, 39). For instance, native (pre-contrast agent injection) and post-contrast agent injection T1 relaxation times of the myocardium and hematocrit constitute the terms of a simple equation to estimate the extracellular volume (ECV), a metric of the non-vascular extra-cellular compartment of the myocardium reflecting the cardiac interstitium (40, 41).

A meta-analysis of 890 patients recovering from severe or mild COVID-19, found that nearly half of convalescing COVID-19 patients had one or more abnormal findings on CMR; the most common among those was an increased native T1 value of the myocardium (26% of cases) (42). Interestingly, the prevalence of abnormal CMR findings was higher in patients with non-determined cTn as compared to those with normal troponin values. This meta-analysis also revealed a high heterogeneity among the original studies, discussed here considering newer findings (Supplementary Table 1), likely reflecting differences in study populations, disease severity, study design, interval time from COVID-19 diagnosis to CMR and variability in scan protocols.

### Hospitalized Patients

Knight et al. (43) reported an identifiable cause of myocardial injury in 69% of cases, with only 3% RV or LV dysfunction, when CMR was carried out in the early aftermath of the acute infection (approximately 1–2 months) in a selected cohort of 29 patients with an unexplained increase of cTn during hospitalization. These findings were corroborated in a subsequent multi-center study by the same group on 148 convalescent COVID-19 patients (all hospitalized with increased cTn, 32% requiring ventilatory support) undergoing CMR approximately two months after the acute phase (44). CMR identified at least one cardiac abnormality in 54% patients which was deemed as ischemic-related in 22%. Non-ischemic (including myocarditis-like pattern), or dual pathology abnormalities were identified in 27% and 5%, respectively. Pericardial effusion was observed in 5% of patients. Native T1 and T2 values were higher in the convalescent COVID-19 patients than in healthy volunteers, though, no statistically significant differences were seen in age, sex, and comorbidity matched controls. This study provided, for the first time, an insight into the cardiovascular abnormalities in a high-risk cohort of hospitalized COVID-19 patients (mean age, 64 years) with multiple risk factors and comorbidities (32% had a diagnosis of pulmonary embolism during hospitalization, 34% were diabetic and 12% had prior coronary revascularization).

Equally a study on 58 hospitalized patients at 2–3 months after infection reported 26% of cases with elevated native myocardial T1 value and 12% of cases with myocarditis pattern by LGE (45). Notably, 83% of patients had at least one cardiopulmonary symptom but only 8% had elevated cTn at the time of CMR. At the 6-month follow-up, cardiac findings normalized so that T1 and LGE prevalence did not differ from a control group matched for cardiovascular risk factors (46). Wu et al. studied 27 hospitalized COVID-19 patients that were symptomatic at the 6-month CMR follow-up, of which, nearly half had elevated cTn and 30% had myocardial scars (47). High incidence of other cardiac abnormalities like elevated ECV and reduced global longitudinal strain (GLS) at 6-months were reported by Li et al. in a cohort of hospitalized COVID-19 patients with no prior cardiovascular disease and with normal cTn and natriuretic peptide (48). These studies were conducted in highly selected populations of hospitalized COVID-19 patients with increased circulating cardiac biomarkers which prevent extrapolation of these findings to the much more prevalent population of convalescent COVID-19 patients with an asymptomatic or milder viral illness.

### Convalescence From Mild or Asymptomatic COVID-19

In a prospective cohort of 100 moderate-to-severe risk (33% hospitalized) convalescent COVID-19 patients, Puntmann et al. (49, 50) reported that as many as 78 patents had cardiovascular involvement when CMR was carried out 2–3 months after the initial infection. Remarkably, native T1 and T2 values were raised in 73% and 60% of patients, respectively, likely reflecting diffuse myocardial inflammation. These metrics also provided the best discrimination for myocardial involvement when tested against healthy and matched controls. Unlike prior studies, Puntmann et al. showed that cardiac involvement was unrelated to the severity of initial COVID-19 presentation and persisted well beyond the acute phase, raising concern about myocardial inflammatory burden in convalescent COVID-19 patients with ongoing breathlessness and fatigue, and the potential for untoward clinical outcomes (51, 52). These findings were in line with later studies reporting elevated T1 or T2 values in convalescent COVID-19 patients as compared to healthy volunteers (48, 53), albeit Puntmann et al. found higher prevalence, which may be due in part to the low bar on the T1 increase necessary to register as “elevated” (a 1.4% increase versus a range of 2.1–3.5% in other papers).

By contrast, in a nested case-control study including 74 seropositive and 75 age-, sex-, ethnicity-matched seronegative controls undergoing comprehensive CMR six months after infection, Joy et al. (54) showed that LV structure, systolic function, myocardial tissue features were similar between cases and controls. The authors concluded that cardiovascular abnormalities are infrequent in convalescent low-risk COVID-19 patients with favorable comorbidity profile (average age 37 years, no antecedent history of heart disease, only 2% and 9% were diabetic and hypertensive, respectively). However, the myocardial T1 was elevated in 8% of cases despite normal circulating cardiac biomarkers, including cTn. In the largest study of 201 mainly non-hospitalized, low risk COVID-19 patients with persistent symptoms the prevalence of cardiac abnormalities on CMR was 26% overall four months after infection (55). In particular, 19% of patients had at least three myocardial segments with elevated native T1 and values were significantly elevated in patients reporting more severe symptoms.

### Convalescent Athletes With COVID-19

To date studies on convalescent athletes recovering from COVID-19 have been limited to short time periods of convalescence, 15–32 days since initial symptoms (56–62). The study by Brito et al. on a young cohort of athletes (median age 19 years, range from 19 to 21 years) was the only one that included matched controls and revealed subclinical myocardial or pericardial disease in 27/48 athletes by CMR but no findings were different to matched controls and only one patient presented accompanying elevation in cTnI (61).

## Impact of Vaccination

There have recently been reports of acute myocarditis cases following anti-COVID vaccination with spike protein mRNA. Among a large Israeli population who had received at least one dose of the BNT162b2 mRNA vaccine, the incidence of myocarditis was 2.13 cases per 100,000 persons (63, 64), exceeding the myocarditis rate in the general population before the COVID-19 outbreak. Similar data in the US reveal by CMR an increased incidence of acute myocarditis (8 per 100,000 persons) after the second dose for mRNA-1273 and the first dose for BNT162b2-mRNA (65). While 65–97% acute myocarditis cases self-resolved (63–66), long-term follow-up data are still lacking, particularly in convalescent COVID-19 patients or other potentially susceptible groups, urging for continued monitoring.

## CMR in a Multi-Organ Damage Context

As with the lung and cardiovascular system, brain, kidneys and liver are vulnerable to virus-mediated injury (24–26, 67–69). Autopsies have shown that SARS-CoV-2 may cause changes in brain parenchyma and vessels, potentially altering the integrity of the blood–brain and blood-cerebrospinal-fluid barriers (70). A large study conducted in nearly 5,500 hospitalized patients in New York city revealed that 37% developed persisting kidney injury (27). Myocardial injury, kidney, hepato-biliary system, and brain involvement in hospitalized COVID-19 patients is associated with worse clinical outcome (71). Importantly, these abnormalities and organ-specific symptoms appear to persist well beyond the acute phase of the disease contributing to the prismatic nature of long covid syndrome.

To date, three studies have investigated cardiac damage alongside other organ damage in COVID-19 survivors using magnetic resonance imaging (MRI) (45, 55). At 2–3 months after first symptoms Raman et al. identified 26% cardiac abnormalities persisting alongside 60% lung abnormalities in a cohort of 58 hospitalized patients (45). As underpinned by an increased T1 relaxation time of organ parenchyma, 10 and 29% of the patients had liver and kidney inflammation, respectively (45). Brain MRI findings suggested higher burden of brain microvascular damage in the convalescent COVID-19 patients as compared to age, sex, risk-factor and BMI-matched controls. In a follow-up study, 52% of the patients remained symptomatic and continued to present heart and lung abnormalities by CMR at 6-month after the acute illness (46). However, RV function, T1 elevation and cardiopulmonary symptoms had improved in comparison to the controls, whilst lung parenchymal abnormalities had not.

In a study of 201 mainly non-hospitalized patients with persisting symptoms, Dennis et al. identified abnormalities by multi-organ MRI in the heart, kidney, liver, pancreas, spleen, and lungs, four months after acute infection (55). Heart, liver, kidney, and pancreas fibroinflammation as revealed by elevated T1 relaxation time on parametric images was found in 19%, 12%, 4%, and 15% of patients, respectively. The liver and pancreas abnormalities were more common in hospitalized patients (55). In 443 mainly non-hospitalized patients, Petersen et al. identified significant cardiovascular (reduced LV ejection fraction) and neurological (higher mean cortical thickness) abnormalities by MRI 10-months after infection, compared to 1,328 matched controls (72).

These findings in convalescent COVID-19 patients warrant a deeper investigation of the relationships between cardiac injury, ongoing symptoms and the influence of pre-existing risk factors and damage to other organs.

## Discussion

Altogether these CMR studies report a cardiovascular finding in 16–83% of convalescent COVID-19 patients at 1–10 months after the acute phase. Individuals with initially asymptomatic or mild COVID-19, not requiring hospitalization appear to have a lower rate of abnormal findings by CMR that have accompanying elevation in cTn compared to patients with initially severe COVID-19 requiring hospitalization (16).

**FIGURE 1:**
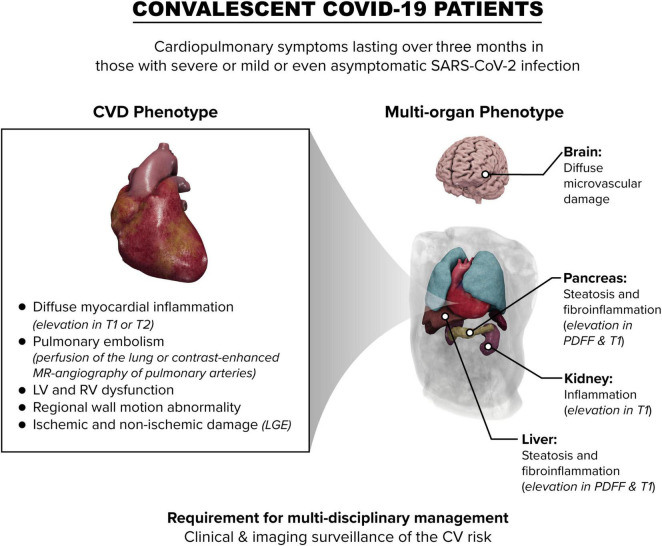
Schematic overview of the impact of COVID-19 on the heart and other organs in convalescent patients. LV, left ventricle; RV right ventricle; CV cardiovascular; LGE, late gadolinium enhancement; PDFF, proton density fat fraction.

The main pathological features of COVID-19 mediated myocardial damage in convalescing patients often differ from classical viral-mediated myocarditis particularly in subjects with less severe disease (73) (Figure 1). Characteristics of acute phase COVID-19 like pericarditis are infrequently observed (74). Thus, the adoption of the Lake Louise criteria applied extensively for myocarditis definition (38, 39), may not always be suitable in the setting of convalescent COVID-19.

Myocardial T1 and T2 relaxation times can be affected by factors like age, sex and patients’ risk factors as well as tissue composition along with several technical aspects relating to the CMR acquisition sequence (75, 76). Increased T1 or T2 relaxation time may not necessarily associate with LGE when increment of intra- and extracellular fluid content in the inflamed tissue is proportional (77, 78). Thus, caution is required when interpreting T1 and T2 changes in the absence of pathological correlates: these should be considered as potentially ongoing myocardial abnormalities whose clinical correlates remain unknown and warrant closer follow-up.

The choice of the control group to diagnose and monitor the COVID-19 mediated cardiac changes is a crucial aspect in future investigations of the long-term consequences of COVID-19. About 8% of asymptomatic subjects with cardiovascular risk factors undergoing CMR had an unexpected myocardial scar (79). Some studies on convalescent COVID-19 patients that included a control group showed that the differences in cardiovascular abnormalities observed between patients and healthy controls were attenuated or became non-significant when the comparisons were more strictly matched by age, sex and cardiovascular risk factors (44, 49, 54). Longitudinal follow-up of patients from earlier studies, with a view to deciphering the relationship between patient symptoms and organ abnormality will aid in this endeavor.

It can be concluded that, given the emerging evidence of both cardiac and multi-organ damage due to SARS-CoV-2 infection in both acute and post-acute disease phases, improved multidisciplinary management of people suffering from continued symptoms is needed.

## Author Contributions

AB, HT-B, and P-GM contributed to the initial design of the article, assorted information, and drafted the manuscript. DF, GG, RB, and MR made a critical revision of the article. HT-B and P-GM governed the whole process. All authors contributed to the article and approved the submitted version.

## Conflict of Interest

AB, HT-B, MR, RB, and PG-M are employees of Perspectum Ltd., a privately-funded commercial enterprise that develops medical devices to address unmet clinical needs, including CoverScan™. The remaining authors declare that the research was conducted in the absence of any commercial or financial relationships that could be construed as a potential conflict of interest.

## Publisher’s Note

All claims expressed in this article are solely those of the authors and do not necessarily represent those of their affiliated organizations, or those of the publisher, the editors and the reviewers. Any product that may be evaluated in this article, or claim that may be made by its manufacturer, is not guaranteed or endorsed by the publisher.
