# Supplementary material for: The Additive Value of Cardiovascular Magnetic Resonance in Convalescent COVID-19 Patients
Source: Front Cardiovasc Med. 2022 Apr 7;9:854750. doi: 10.3389/fcvm.2022.854750 (PMC9021393; doi:10.3389/fcvm.2022.854750)
Supplement: Supplementary file 1 [file Table_1.docx]

**The additive value of cardiovascular magnetic resonance in convalescent COVID-19 patients**

Supplementary materials

Table 1. Prevalence of cardiovascular abnormality in convalescent COVID-19. Studies ordered by increasing time since first symptoms. Studies with 100% patients hospitalized for COVID-19 shown in **bold**.

| First Author, year | Study design | Population characteristics | Cohort n  (Patients:  Matched Controls:  Healthy controls) | Cardiac biomarkers | CMR acquisition | MRI phenotype | CMR findings |
| --- | --- | --- | --- | --- | --- | --- | --- |
| CMR 2 to 8 weeks from symptoms | | | | | | | |
| **Maurus, 2021** (1)  **Weckbach, 2021** (2) | Prospective study  1 German center  CMR on inpatients with modestly elevated hsTnI or suspected myocarditis within mean 22±13 days after initial symptoms (PCR or clinical diagnosis) | Patients:  -Mean age 76±4 yr,  -89% male  -33% prior heart disease  -100% hospitalized with COVID-19  -100% continued symptoms at CMR visit | 18:18:0 | Elevated hscTnI at CMR visit | 1.5T  cine  T2W  Native and post contrast T1 map (MOLLI)  T2 map  LGE | Mapping: 16 AHA segments model (thresholds from healthy volunteers)  2018 LLC to assess myocarditis  T2W signal intensity ratio | 56% elevated T1  39% myocardial edema  33% myocarditis  11% reduced LVEF  67% LGE positive  22% pericardial effusion  33% myocarditis  Total any CMR finding: 83% |
| Brito, 2021 (3) | Prospective study  1 US center  CMR on college athletes still symptomatic or with abnormal ECG or TEE within median 27 days after initial symptoms (PCR or antibody test) | Patients:  -Mean age 19 yr (range 18-21)  -85% male, 26% white,  -0% hospitalized with COVID-19 | 48:20:16 | 1 patient with elevated hscTnI at CMR visit | 1.5T  cine  Native and post contrast T1 map (ShMOLLI)  T2 map  T2W  LGE | Mapping: ROI at septum in mid or basal cavity and individual non-apical segments  T2W signal intensity ratio | 19% elevated myocardial T1  0% elevated myocardial T2  40% pericardial enhancement  2% LGE positive  2% with reduced EF  Total any CMR finding: 56% |
| **Huang, 2020** (4) | Retrospective study  1 China center  CMR on discharged hospital patients referred for CMR within median 47 days after initial symptoms (PCR diagnosis and cardiac symptoms) and median | Patients:  -Median age 38 yr (range 32-45),  -38% male  -0% prior heart disease  -100% hospitalized with COVID-19 | 26: 20: 0 | 0% elevated hscTnI at CMR visit | 3T  cine  Native and post-contrast T1 map (MOLLI)  T2 map  T2W  LGE | Mapping: reference ROI placed in a remote, non-enhanced area of myocardium  T2W signal intensity ratio  LGE 5SD | 58% increased T2 signal and/or LGE positive  4% reduced LVEF  27% pericardial effusion |
| **Pan, 2021** (5) | Prospective study  1 China center  CMR on discharged hospital patients without clinical or serological evidence of myocardial injury within median 46 (range 43-50) after hospitalisation | Patients:  -Median age 36 (31-47) yr  -48% male  -0% prior heart disease  -100% hospitalized with COVID-19 | 21:20:0 | Normal hscTnI and BNP at hospitalisation and at CMR visit | 3T  cine  T2W  Native T1 map (MOLLI)  T2 map | Mapping: average of 16 AHA segments  (normal thresholds based on matched controls)  T2W signal intensity ratio | 0% oedema on T2W  24% elevated T1  48% elevated T2  5% reduced LVEF  10% reduced RVEF  Total any CMR finding: 71% |
| **Knight, 2020** (6) | Prospective study  1 UK center  CMR on discharged hospital patients with elevated hsTnI within mean 46±15 days after initial symptoms (PCR or clinical diagnosis or chest imaging) | Patients:  -mean age 64±9 yr  -83% male  -100% hospitalized with COVID-19, (32% at ICU) | 29:0:0 | Elevated hsTnT at hospitalization | 1.5T  cine  Native T1 map  T2 map  EGE and LGE  Stress perfusion (66%) | LGE visual assessment  2018 LLC to assess myocarditis | 3% mild LV dysfunction  3% severe LV and RV dysfunction  7% pericardial effusion  45% myocarditis-like pattern by LGE |
| \| CMR 8 weeks to 4 months from symptoms \| \| --- \| | | | | | | | |
| **Raman, 2021** (7) | Prospective study  1 UK center  CMR and multiorgan MRI on discharged hospital patients within median 69 days after initial symptoms (PCR diagnosis) and median 48 days after discharge | Patients:  -Mean age 55 ±13 yr,  -59% male  5% prior heart disease  -100% hospitalized with COVID-19 (36% at ICU)  -64% continued symptoms at CMR visit | 58:30:0 | 0% elevated hsTnI at CMR visit  20% elevated NT-proBNP at CMR visit | 3T  cine  Native and post contrast T1 map (ShMOLLI)  T2 map  LGE | LGE 5SD  Mapping: base, mid and apical slices and average slices  Reference ROI placed in a remote, non-enhanced area of myocardium | 26% elevated basal T1  8% elevated mid T1  2% elevated apical T1  6% elevated basal T2  2% elevated mid T2  2% elevated apical T2  2% pericardial effusion  12% myocarditis pattern by LGE |
| **Kotecha, 2021** (8) | Prospective study  6 UK centers  CMR on discharged hospital patients with elevated hsTnI referred for CMR within median 68 days after initial symptoms (PCR or chest imaging) and median 56 days after discharge | Patients:  -Mean age 64 ±12 yr  -70% male  -19% prior heart disease  -100% hospitalized with COVID-19 (32% at ICU) | 148:40:40 | Elevated hscTnI at hospitalisation | 1.5T  cine  Native T1 map (MOLLI)  T2 map  LGE | Mapping: ROI in the septum in both remote and disease areas | 3% elevated septal T2  13% elevated septal T1  3% elevated remote T2  14% elevated remote T1  49% LGE positive  27% clinical myocarditis based on all CMR findings  5% pericardial effusion  11% reduced LVEF (<55%)  Total any CMR finding: 54% |
| **Wang, 2021** (9) | Prospective study  1 China center  CMR on discharged hospital patients within mean 103±21 days after hospital discharge | Patients:  -Mean age 48±13 yr  -43% male  -0% prior heart disease  -100% hospitalized with COVID-19 | 44:31:0 | HscTnI measured | 3T  cine  Native and post contrast T1 map (MOLLI)  T2W  T2*map  LGE | LGE FWHM  Strain using tissue tracking (GLS, GCS, GRS)  Global T1 | 30% LGE positive, (this group had decreased LV GCS and RV strain) |
| **Urmeneta Ulloa, 2021** (10) | Prospective study  1 Spanish center  CMR on discharged patients with dyspnoea or chest pain and/or transthoracic echocardiogram (TTE) (LV dysfunction/alterations in segmental contractility, suspected right ventricle (RV) dilatation/dysfunction) or ECG alterations within 81 ± 27 days from contact | Patients:  -mean age 59 ± 15 years,  -80.7% male  -0% prior heart disease  -100% hospitalized with Covid-19 (7% at ICU) | 57:0:20 |  | 1.5T  cine  Native and post contrast T1 map (MOLLI)  T2W  T2 map  LGE | Mapping: ROI in midventricular septum  LGE and T2W visual assessment | 19% non ischemic LGE positive  4% ischemic LGE positive  4% pericardial LGE positive  11% oedema by T2W |
| Puntmann, 2020 (11) | Prospective study  1 German center  CMR on any patient within median 71 days after initial COVID symptoms (PCR or clinical diagnosis) | Patients:  -Mean age 49 ±14 yr  -53% male  -13% prior heart disease  -33% hospitalized with COVID-19, 36% continued fatigue or breathlessness at CMR visit | 100:57:50 | 5% had elevated hsTnT at CMR | 3T  Cine  Native T1 map (MOLLI)  T2 map  LGE | Mapping: ROI in the septum (area of LGE excluded) | 73% elevated T1  60% elevated T2  32% with any myocarditis by LGE  20% non-ischemic myocarditis by LGE  6% reduced LVEF  7% reduced RVEF  13% elevated LVEDVI  Total any CMR finding: 78% |
| Eiros, 2020 (12) | Prospective study  1 Spanish center  CMR were performed 10.4 (9.3–11.0) weeks after symptoms of infection | Patients: health care workers.  -Median age 52 (41–57) yr  -28% male  -6% prior heart disease  -16% hospitalized | 139:0:20 | 8% had elevated NT-pro-BNP and 1% had elevated Troponin at CMR visit | 1.5T  cine  T2W  Native and post contrast T1 map  T2 map  LGE | Mapping: 16 AHA segments model  Strain by feature tracking analysis  2018 LLC to assess myocarditis | 14% pericarditis  37% myocarditis  11% both pericarditis and myocarditis  4% elevated T2  4% oedema on T2W  42% elevated native T1  37% elevated ECV  7% LGE positive  30% pericardial effusion  5% systolic left ventricular wall motion abnormalities, global or regional  Overall 75% CMR abnormalities |
| Breitbart, 2021 (13) | Prospective study  1 German center  CMR were performed on patients referred for persistent symptoms such as chest pain/ discomfort, shortness of breath, or intolerance to activity at 77±66 days after COVID-19 disease | Patients:  -mean age 46±12 years,  -46% male  -0% prior heart disease  -9% hospitalized  -100% continued symptoms at CMR visit | 56:0:0 | 2% had elevated NTpro-BNP at CMR visit | 1.5T  cine  Native and post contrast T1 map (MOLLI)  T2 map  LGE | Mapping: 16 AHA segments analysis (2SD above internal references was defined as abnormal)  2018 LLC to assess myocarditis | 12% LGE positive  2% elevated T1  5% elevated T2  2% fulfil LLC criteria  16% Overall CMR abnormalities |
| Kravchenko, 2021 (14) | Prospective study  1 German center  CMR were performed on non-hospitalized patients with symptoms 103 days (interquartile range: 88-158). Troponin T levels were normal. | Patients:  -mean age 39±13 years;  -44% male  -0% prior heart disease  -0% hospitalized  -100% continued symptoms at CMR visit | 41:0:42 | Normal Troponin T level | 1.5T  cine  T2W  Native and post contrast T1 map (MOLLI)  T2 map  LGE | LGE FWHM  T2W signal intensity ratio  Mapping: global (thresholds for abnormality)  2018 LLC to assess myocarditis | 7% non-ischemic LGE  0% myocardial oedema  2% pericardial effusion  0% fulfil LLC criteria |
| \| CMR more than 4 months from symptoms \| \| --- \| | | | | | | | |
| **Wu, 2021** (15) | Prospective study  1 China center  CMR on discharged hospital patients at 180 days after discharge (6-month follow-up) | Patients:  -Median age 63 (range 58-70) yr  -54% male  -100% hospitalized with COVID-19  -100% continued symptoms at CMR visit | 27:0:0 | 48% Elevated hscTnI at hospitalisation | 3T  cine  Native and post contrast T1 map (MOLLI)  LGE | Not described | 30% LGE positive |
| **Li, 2021** (16) | Prospective study  1 China center  CMR on discharged hospital patients with mean 158±18 after hospitalization and mean 124±17 days after hospital discharge | Patients:  -Mean age 54±12 yr,  -60% male,  -0% prior heart disease  -100% hospitalized with COVID-19 | 40:25:0 | Normal cTnI and NTpro-BNP | 3T  cine  Native and post contrast T1 map (MOLLI)  LGE | LGE 5SD  Global T1  Strain using feature tracking (GLS) | 3% LGE positive  60% elevated ECV  70% reduced GLS |
| **Myhre, 2021** (17) | Prospective study  1 Norwegian center  CMR on discharged patients at median 175 (IQR 105-217) days (range 75-246) from hospital admission | Patients:  -Median age 56 (50-70) yr,  -56% male  -9% cardiovascular disease  -100% hospitalized with COVID-19, (19% at ICU) | 48:0:32 | 28% elevated cTnT  35% elevated NT-proBNP at hospitalisation | 1.5T  cine  Native and post contrast T1 (MOLLI)  T2W  T2 map  LGE | LGE 5SD  T2W visual assessment  Mapping: ROI in midventricular septum  Strain using feature tracking (GLS, GCS) | 21% LGE positive and/or EF<50% |
| Dennis, 2021 (18) | Prospective study  2 UK centers  CMR and multiorgan MRI on any patient within median 141 days after initial symptoms (PCR or clinical diagnosis) | Patients:  -Median age 44 (range 21-71) yr  -29% male  ^-^5% prior heart disease  -19% hospitalized with COVID-19  -96% continued fatigue or breathlessness at CMR visit | 201:0:36 |  | 62% on 1.5T  38% on 3T  cine  Native T1 map (MOLLI) | T1 map: AHA 16 segments model | 19% with >3 segments with elevated T1  5% with reduced LVEF  4% with elevated LVEDV  Total any CMR finding: 26% |
| Joy, 2021 (19) | Prospective study  3 UK centers  CMR on healthcare workers within median 186 days after initial symptoms (PCR and seropositivity) | Patients:  -Median age 39 yr (range 30-48)  -48% male  -0% prior heart disease  -1% hospitalized with COVID-19 -5% continued fatigue or breathlessness at CMR visit | 74:75:0 | 0% elevated NT-proBNP at CMR visit | 1.5T  cine  Native and post contrast T1 map (MOLLI)  T2 map  LGE  Aortic pulse wave velocity | LGE 3SD  Mapping: ROI in the septum and global values  Aortic distensibility at diaphragm | 4% myocarditis-like scarring by LGE  8% LGE positive  9% and 5% elevated septal and global T2  7% and 5% elevated septal and global T1  4% elevated septal and global ECV |
| **Cassar, 2021** (20) | Prospective study  1 UK center  CMR at median of 6 months from disease onset (6-6.8) | Patients:  -Mean age 55 ±13 yr,  -63% male  -2% prior heart disease  -100% hospitalized with COVID-19 (37% at ICU)  -52% continued symptoms at CMR visit | 46:30:0 | 0% elevated hsTnI at CMR visit  17% elevated NT-proBNP at CMR visit | 3T  cine  Native and post contrast T1 map (ShMOLLI)  T2 map  LGE | LGE 5SD  Mapping: base, mid and apical slices and average slices  Reference ROI placed in a remote, non-enhanced area of myocardium | 9% elevated basal T1  2% elevated mid T1  2% elevated apical T1  2% elevated basal T2  2% elevated mid T2  12% myocarditis by LGE  6% elevated basal ECV  5% reduced LVEF |

References

1. Maurus S, Weckbach LT, Marschner C, Kunz WG, Ricke J, Kazmierczak PM, Bieber S, Brado J, Kraechan A, Hellmuth JC, et al. Differences in Cardiac Magnetic Resonance Imaging Markers Between Patients With COVID-19-associated Myocardial Injury and Patients With Clinically Suspected Myocarditis. *Journal of Thoracic Imaging* (2021) **36**:279–285. doi: 10.1097/RTI.0000000000000599

2. Weckbach LT, Curta A, Bieber S, Kraechan A, Brado J, Hellmuth JC, Muenchhoff M, Scherer C, Schroeder I, Irlbeck M, et al. Myocardial Inflammation and Dysfunction in COVID-19–Associated Myocardial Injury. *Circulation: Cardiovascular Imaging* (2021) **14**:e012220–e012220. doi: 10.1161/CIRCIMAGING.120.011713

3. Brito D, Meester S, Yanamala N, Patel HB, Balcik BJ, Casaclang-Verzosa G, Seetharam K, Riveros D, Beto 2nd RJ, Balla S, et al. High Prevalence of Pericardial Involvement in College Student Athletes Recovering From COVID-19. *JACC Cardiovascular imaging* (2021) **14**:541–555. doi: 10.1016/j.jcmg.2020.10.023

4. Huang L, Zhao P, Tang D, Zhu T, Han R, Zhan C, Liu W, Zeng H, Tao Q, Xia L. Cardiac Involvement in Patients Recovered From COVID-2019 Identified Using Magnetic Resonance Imaging. *JACC Cardiovascular imaging* (2020) **13**:2330–2339. doi: 10.1016/j.jcmg.2020.05.004

5. Pan C, Zhang Z, Luo L, Wu W, Jia T, Lu L, Liu WV, Qin Y, Hu F, Ding X, et al. Cardiac T1 and T2 Mapping Showed Myocardial Involvement in Recovered COVID-19 Patients Initially Considered Devoid of Cardiac Damage. *Journal of Magnetic Resonance Imaging : JMRI* (2021)10.1002/jmri.27534-10.1002/jmri.27534. doi: 10.1002/jmri.27534

6. Knight D, Tushar K, Yousuf R, Liza C, T. BJ, S. JP, James G, Michael J, E. LL, Rupert N, et al. COVID-19 Myocardial Injury in Survivors. *Circulation* (2020) **142**:1120–1122. doi: 10.1161/CIRCULATIONAHA.120.049252

7. Raman B, Cassar MP, Tunnicliffe EM, Filippini N, Griffanti L, Alfaro-Almagro F, Okell T, Sheerin F, Xie C, Mahmod M, et al. Medium-term effects of SARS-CoV-2 infection on multiple vital organs, exercise capacity, cognition, quality of life and mental health, post-hospital discharge. *EClinicalMedicine* (2021) **31**:100683–100683. doi: 10.1016/j.eclinm.2020.100683

8. Kotecha T, Knight DS, Razvi Y, Kumar K, Vimalesvaran K, Thornton G, Patel R, Chacko L, Brown JT, Coyle C, et al. Patterns of myocardial injury in recovered troponin-positive COVID-19 patients assessed by cardiovascular magnetic resonance. *European Heart Journal* (2021) **42**:1866–1878. doi: 10.1093/eurheartj/ehab075

9. Wang H, Li R, Zhou Z, Jiang H, Yan Z, Tao X, Li H, Xu L. Cardiac involvement in COVID-19 patients: mid-term follow up by cardiovascular magnetic resonance. *Journal of Cardiovascular Magnetic Resonance* (2021) **23**:14–14. doi: 10.1186/s12968-021-00710-x

10. Urmeneta Ulloa J, Martínez de Vega V, Salvador Montañés O, Álvarez Vázquez A, Sánchez-Enrique C, Hernández Jiménez S, Sancho García FD, López Ruiz L, Recio Rodríguez M, Pizarro G, et al. Cardiac magnetic resonance in recovering COVID-19 patients. Feature tracking and mapping analysis to detect persistent myocardial involvement. *IJC Heart & Vasculature* (2021) **36**:100854. doi: 10.1016/j.ijcha.2021.100854

11. Puntmann VO, Carerj ML, Wieters I, Fahim M, Arendt C, Hoffmann J, Shchendrygina A, Escher F, Vasa-Nicotera M, Zeiher AM, et al. Outcomes of Cardiovascular Magnetic Resonance Imaging in Patients Recently Recovered From Coronavirus Disease 2019 (COVID-19). *JAMA Cardiology* (2020) **5**:1265–1273. doi: 10.1001/jamacardio.2020.3557

12. Eiros R, Barreiro-Perez M, Martin-Garcia A, Almeida J, Villacorta E, Perez-Pons A, Merchan S, Torres-Valle A, Pablo CS, González-Calle D, et al. Pericarditis and myocarditis long after SARS-CoV-2 infection: a cross-sectional descriptive study in health-care workers. (2020). doi: 10.1101/2020.07.12.20151316

13. Breitbart P, Koch A, Schmidt M, Magedanz A, Lindhoff-Last E, Voigtländer T, Schmermund A, Mehta RH, Eggebrecht H. Clinical and cardiac magnetic resonance findings in post-COVID patients referred for suspected myocarditis. *Clin Res Cardiol* (2021) doi: 10.1007/s00392-021-01929-5

14. Kravchenko D, Isaak A, Zimmer S, Mesropyan N, Reinert M, Faron A, Pieper CC, Heine A, Velten M, Nattermann J, et al. Cardiac MRI in Patients with Prolonged Cardiorespiratory Symptoms after Mild to Moderate COVID-19. *Radiology* (2021) **301**:E419–E425. doi: 10.1148/radiol.2021211162

15. Wu X, Deng K-Q, Li C, Yang Z, Hu H, Cai H, Zhang C, He T, Zheng F, Wang H, et al. Cardiac Involvement in Recovered Patients From COVID-19: A Preliminary 6-Month Follow-Up Study. *Frontiers in cardiovascular medicine* (2021) **8**:654405–654405. doi: 10.3389/fcvm.2021.654405

16. Li X, Wang H, Zhao R, Wang T, Zhu Y, Qian Y, Liu B, Yu Y, Han Y. Elevated Extracellular Volume Fraction and Reduced Global Longitudinal Strains in Participants Recovered from COVID-19 without Clinical Cardiac Findings. *Radiology* (2021) **299**:E230–E240. doi: 10.1148/radiol.2021203998

17. Myhre PL, Heck SL, Skranes JB, Prebensen C, Jonassen CM, Berge T, Mecinaj A, Melles W, Einvik G, Ingul CB, et al. Cardiac pathology 6 months after hospitalization for COVID-19 and association with the acute disease severity. *Am Heart J* (2021) **242**:61–70. doi: 10.1016/j.ahj.2021.08.001

18. Dennis A, Wamil M, Alberts J, Oben J, Cuthbertson DJ, Wootton D, Crooks M, Gabbay M, Brady M, Hishmeh L, et al. Multiorgan impairment in low-risk individuals with post-COVID-19 syndrome: a prospective, community-based study. *BMJ Open* (2021) **11**:e048391–e048391. doi: 10.1136/bmjopen-2020-048391

19. Joy G, Artico J, Kurdi H, Seraphim A, Lau C, Thornton GD, Oliveira MF, Adam RD, Aziminia N, Menacho K, et al. Prospective Case-Control Study of Cardiovascular Abnormalities 6 Months Following Mild COVID-19 in Healthcare Workers. *JACC: Cardiovascular Imaging* (2021) **14**:2155–2166. doi: 10.1016/j.jcmg.2021.04.011

20. Cassar MP, Tunnicliffe EM, Petousi N, Lewandowski AJ, Xie C, Mahmod M, Samat AHA, Evans RA, Brightling CE, Ho L-P, et al. Symptom Persistence Despite Improvement in Cardiopulmonary Health - Insights from longitudinal CMR, CPET and lung function testing post-COVID-19. *EClinicalMedicine* (2021) **41**:101159. doi: 10.1016/j.eclinm.2021.101159
